# ATC-NLSP: Prediction of the Classes of Anatomical Therapeutic Chemicals Using a Network-Based Label Space Partition Method

**DOI:** 10.3389/fphar.2019.00971

**Published:** 2019-09-05

**Authors:** Xiangeng Wang, Yanjing Wang, Zhenyu Xu, Yi Xiong, Dong-Qing Wei

**Affiliations:** State Key Laboratory of Microbial Metabolism, School of Life Sciences and Biotechnology, Shanghai Jiao Tong University, Shanghai, China

**Keywords:** drug classification, multilabel classification, label correlation, label space partition, label propagation

## Abstract

Anatomical Therapeutic Chemical (ATC) classification system proposed by the World Health Organization is a widely accepted drug classification scheme in both academic and industrial realm. It is a multilabeling system which categorizes drugs into multiple classes according to their therapeutic, pharmacological, and chemical attributes. In this study, we adopted a data-driven network-based label space partition (NLSP) method for prediction of ATC classes of a given compound within the multilabel learning framework. The proposed method ATC-NLSP is trained on the similarity-based features such as chemical–chemical interaction and structural and fingerprint similarities of a compound to other compounds belonging to the different ATC categories. The NLSP method trains predictors for each label cluster (possibly intersecting) detected by community detection algorithms and takes the ensemble labels for a compound as final prediction. Experimental evaluation based on the jackknife test on the benchmark dataset demonstrated that our method has boosted the absolute true rate, which is the most stringent evaluation metrics in this study, from 0.6330 to 0.7497, in comparison to the state-of-the-art approaches. Moreover, the community structures of the label relation graph were detected through the label propagation method. The advantage of multilabel learning over the single-label models was shown by label-wise analysis. Our study indicated that the proposed method ATC-NLSP, which adopts ideas from network research community and captures the correlation of labels in a data driven manner, is the top-performing model in the ATC prediction task. We believed that the power of NLSP remains to be unleashed for the multilabel learning tasks in drug discovery. The source codes are freely available at https://github.com/dqwei-lab/ATC.

## Introduction

The Anatomical Therapeutic Chemical (ATC) Classification System (MacDonald and Potvin, 2004), maintained by the World Health Organization Collaborating Centre for Drug Statistics Methodology, is the most widely accepted and canonical scheme for drug categorization. This system assigns different group labels for drugs based on the organ or systems where they take effect and/or their therapeutic, pharmacological, and chemical attributes. The ATC system is a strict hierarchy, including five levels of classification, and for the first level, there are 14 main groups: 1) alimentary tract and metabolism (coded by **A**); 2) blood and blood-forming organs (coded by **B**); 3) cardiovascular system (coded by **C**); 4) dermatologicals (coded by **D**); 5) genitourinary system and sex hormones (coded by **G**); 6) systemic hormonal preparations, excluding sex hormones and insulins (coded by **H**); 7) anti-infectives for systemic use (coded by **J**); 8) antineoplastic and immunomodulating agents (coded by **L**); 9) musculoskeletal system (coded by **M**); 10) nervous system (coded by **N**); 11) antiparasitic products, insecticides, and repellents (coded by **P**); 12) respiratory system (coded by **R**); 13) sensory organs (coded by **S**); and 14) various (coded by **V**). Given a new compound, prediction of its ATC classes can provide us with deeper insights into its therapeutic indications and side effects, thus accelerating both basic research and drug development (Hutchinson et al., 2004; Dunkel et al., 2008).

Traditionally, identification of ATC classes for a new drug using experimental methods is both time- and resource-consuming. Therefore, *in silico* prediction of ATC classes of a compound by machine learning techniques is a hot field in drug discovery and development. Previous studies (Dunkel et al., 2008; Wu et al., 2013) formulate the prediction of ATC classes as a single-label learning task, which is suggested to be inappropriate due to the multilabel nature of this biological system (Chou, 2013). Within the multilabel learning framework, Cheng et al. (2017b) proposed a multilabel predictor iATC-mISF, which utilized multilabel Gaussian kernel regression and three types of features (chemical–chemical interaction, structural similarity, and fingerprint similarity). The iATC-mISF has been upgraded as iATC-mHyb (Cheng et al., 2017a) by further incorporating drug ontological information. Besides one-dimensional representation of features, inspired by the histograms of oriented gradients (HoG) method proposed by the computer vison community (Dalal and Triggs, 2005), Nanni and Brahnam (2017) reshaped the features into two-dimensional matrix and performed slightly better than iATC-mISF. Continuing in this direction, the same group (Lumini and Nanni, 2018) applied pretrained convolutional neural networks models on the two-dimensional feature matrix as a featurizer and achieved best performance among the previously published methods on this task.

Typically, multilabel (ML) classification algorithms are classified into three major groups: algorithm adaptation, problem transformation, and ensembles of multilabel classifier (EMLC) (Wan et al., 2017). Algorithm adaptation methods incorporate specific tricks that modify traditional single-label learning algorithms into multilabel ones. The representative algorithm of this group is ML-*k*NN (Zhang and Zhou, 2005). For the problem transformation method, it converts multilabel learning problem into one or more single-label problems. The common strategies for such a transformation include binary relevance, classifier chains, label ranking, and label powerset (LP) (Read et al., 2011). LP trains models on each possible subset of label sets (Gibaja and Ventura, 2014). For a dataset with high cardinality in the large label set, LP is prone to be overfitting because of the exponentially increased number of subsets. To tackle the overfitting nature of label powerset, (Tsoumakas et al., 2011) proposed the RA*k*EL*d* method, which divides the label set into *k* disjoint subsets and use label powerset in these subsets. One major drawback of RA*k*EL*d* is that the *k* is arbitrarily chosen without incorporating the label correlations, which can be possibly learnt from the training data. The **n**etwork-based **l**abel **s**pace **p**artition (NLSP) (Szymański et al., 2016) is an EMLC built upon ML. This NLSP method divides the label set into *k* small-sized label sets (possibly intersecting) by a community detection method, which can incorporate the label correlation structures in the training set, such that it finally learns *k* representative ML classifiers. As a result, NLSP tackles much less subsets compared to LP on the original label set and selects *k* in a data-driven manner. For more detailed explanation of multilabel learning, refer to (Zhang and Zhou, 2014; Moyano et al., 2018).

In this study, we adopted an NLSP method to explore the correlation among labels. Our NLSP method was evaluated on a benchmark dataset (Chen et al., 2012) by the jackknife test. The proposed method demonstrates its superiority over other state-of-the-art approaches by our experimental results. The main strength of our method hinges on two aspects. On the one hand, the NLSP clusters the label space into subspaces and utilizes the correlation among labels. On the other hand, the ensemble learning nature of NLSP on the overlapping subspace could further improve model performance. Interesting patterns on the label relation graph were also detected by NLSP. In addition, the label-wise analysis of the best NLSP model was performed to provide experimental biologists with more insights.

## Materials and Methods

### Benchmark Dataset and Sample Formulation

We utilized the same dataset as the previous study (Cheng et al., 2017b) to facilitate model comparison. This dataset consists of 3,883 drugs, and each drug is labeled with at least one or more of 14 main ATC classes. It is a tidy dataset where no missing value and contradictory record. The UpSet visualization technique (Lex et al., 2014) was used for quantitative analysis of interactions of label sets.

Then, we adopted the same method provided by (Cheng et al., 2017b) to represent the drug samples. The dataset can be formulated in set notation as the union of elements in each class: $S=S_{1}\cup S_{2}\ldots \cup S_{14}$ (1), and a sample *D* can be represented by concatenating the following three types of features.

1. A 14-dimentional vector, *D* **^Int^** = [Φ_1_Φ_2_Φ_3_ … Φ_14_]*^T^* (2), which represents its maximum interaction score Φ*_i_* (Kotera et al., 2012) with the drugs in each of the 14 $S_{i}$.
2. A 14-dimentional vector, *D* **^StrSim^** = [Ψ_1_Ψ_2_Ψ_3__…_ Ψ_14_]*^T^* (3) which represents its maximum structural similarity score Ψ*_i_* (Kotera et al., 2012) with the drugs in each of the 14 $S_{i}$.
3. A 14-dimentional vector, *D* **^FigSim^** = [T_1_T_2_T_3_ … T_14_]*^T^* (4), which represents its molecular fingerprint similarity score T*_i_* (Xiao et al., 2013) with the drugs in each of the 14 $S_{i}$.

Therefore, a given drug *D* is formulated by:

(5) $$D=D^{\mathrm{Int}}⊕D^{\mathrm{StrSim}}⊕D^{\mathrm{FigSim}}={\left[{@}_{1}{@}_{2}{@}_{3}\ldots {@}_{42}\right]}^{T}$$

Where ⊕ represents the symbol for orthogonal sum and where

(6) $${@}_{\text{u}}=\{\begin{array}{c} \Phi_{u}(1\leq u\leq 14)\\ \Psi_{u}(15\leq u\leq 28)\\ {\text{T}}_{u}(29\leq u\leq 42) \end{array}$$

For more details, refer to Cheng et al. (2017b).

### Measuring Label Correlation

In order to evaluate the correlation between two labels, we calculated the bias corrected Cramér’s V statistic for all the label pairs (Bergsma, 2013). Cramér’s V (sometimes referred to as Cramér’s phi and denoted as ϕc) statistic is a measure of association between two nominal variables, ranging from 0 to 1 (inclusive). The bias corrected Cramér’s V statistic is given by (here *n* denotes sample size and *χ*^2^ stands for the chi-square statistic without a continuity correction for a contingency table with *r* rows and *c* columns)

(7) $$\tilde{V}=\sqrt{\frac{{\tilde{\varphi }}^{2}}{\tilde{m}}}$$

where

(8), $${\tilde{\varphi }}^{2}=\max(0,\varphi^{2}-\frac{\left(r-1)(c-1\right)}{n-1}))$$

(9) $$\varphi^{2}=\frac{\chi^{2}}{n}$$

and

(10), $$\tilde{m}=\min(\tilde{r}-1,\tilde{c}-1)$$

(11), $$\tilde{r}=r-\left(\frac{{\left(r-1\right)}^{2}}{n-1}\right)$$

(12). $$\tilde{c}=c-\left(\frac{{\left(c-1\right)}^{2}}{n-1}\right)$$

### Network-Based Label Space Partition

The NLSP is a newly proposed multilabel learning method and has achieved top performance in some predictive tasks (Szymański et al., 2016). In this study, we adopted the data-driven NLSP method for prediction of ATC classes of a compound. NLSP divides the predictive modeling task into the training and classification phase.

In the training phase, four steps are preformed:

1. Establishing a label co-occurrence graph on the training set. The label co-occurrence graph *G* has the label set *L* as the vertex set and the edge between two vertices (labels) exists if at least one sample *S* in training set *D* *_train_* is assigned by these two labels *l* *_i_* and *l* *_j_* together (here *l* *_i_*, *l* *_j_* denote labels of the set *L* *_s_*, which stands for the assigned label set of a sample *S*; || || stands for the cardinality of a given set):
  (13) $$E=\left\{\{l_{i},l_{j}\}:(\exists (S,L_{s})\in D_{\mathrm{train}})\left(l_{i}\in L_{s}\wedge l_{j}\in L_{s}\right)\right\}$$
  We can also easily assign weights to *G* by defining a counting function *w*: *L* → ℕ:
  (14) w(li,lj)=number of sample S that have both labels assignedw(li,lj)=‖{S:(S,Ls)∈Dtrain∧li∈Ls∧lj∈Ls}‖
2. Detecting community on the label co-occurrence graph. There are various community detection algorithms. In this study, we utilized the following two methods to identify communities because both of the two methods have linear time complexity:
  - **Largest modularity using incremental greedy search (Louvain method)** (Blondel et al., 2008): This method is based on greedy aggregation of communities, beginning with communities with single convex and merging the communities iteratively. In each step, two communities are merged when the merging makes the highest contribution to modularity. The algorithm halts when there is no merge that could increase current modularity. This method is frequently referred as “Louvain method” in the network research community. The detailed explanation of this method is described in **Supplementary Method S1**.
  - **Multiple async label propagation (LPA)** (Raghavan et al., 2007): This method assigns unique tags to every vertex in a graph and then iteratively updates the tags of every vertex. This update reassigns the tag of the majority of neighbors to the central vertex. The updating order of vertices shuffled at each iteration. The algorithm is stopped when all vertices have tags identical to the dominant tag in proximity. The detailed description of LPA is appended in **Supplementary Method S2**.
3. For each community *C* *_i_*, corresponding training set *D* *_i_* is created by taking the original dataset with label columns presented in *L* *_i_*.
4. For each community, a base predictor *b* *_i_* is learnt on the training set *D* *_i_*. In this study, we compared the performance of five types of base predictors:
  - **Extremely randomized trees (ERT)** (Geurts et al., 2006; Li et al., 2019) is an ensemble method that adds more randomness compared to random forests by the random top–down splitting of trees instead of computing the locally optimal cut-point for each feature under consideration. This increase in randomness allows to reduce the variance of the model a bit, at the expense of a slightly greater increase in bias.
  - **Random forests (RF)** (Breiman, 2001) is an ensemble method that combines the probabilistic predictions of a number of decision tree-based classifiers to improve the generalization ability over a single estimator.
  - **Support vector machine (SVM)** (Cortes and Vapnik, 1995) is a widely used classification algorithm which tries to find the maximum margin hyperplane to divide samples into different classes. Incorporated by kernel trick, this method could handle both linear and no-linear decision boundary.
  - **Extreme gradient boosting (XGB)** (Chen and Guestrin, 2016) is a newly proposed boosting method, which has achieved state-of-the-art performance on many tasks with tabular training data (Chen et al., 2018). Traditional gradient boosting machine is a meta algorithm to build an ensemble strong learner from weak learners such as decision trees, while XGB is an efficient and distributed implementation of gradient boosting machine.
  - **Multilayer perceptron (MLP)** (Ruck et al., 1990) is a supervised learning algorithm which could learn nonlinear models. It has one or more nonlinear hidden layers between the input and output. For each hidden layer, different numbers of hidden neurons can be assigned. Each hidden neuron yields a weighted linear summation of the values from the previous layer, and the nonlinear activation function is followed. The weights are learnt through backpropagation algorithm or variations upon it.

In the classification phase, we just perform predication on all communities detected in the training phase and fetch the union of assigned labels:

(15) $$b(S)=\cup_{j=1}^{k}b_{i}(S)$$

### Parameter Tuning

There are two layers of hyperparameters tunable for NLSP:

1. The base learner: we chose five types of base learners.
  - Extremely randomized trees: we tuned the hyperparameter of number of trees at [500, 1000], other hyperparameters are at the default values.
  - Random forests: we tuned hyperparameter of number of trees at [500, 1,000], other hyperparameters are at the default values.
  - Support vector machine: we tuned the hyperparameter of *C* (penalty) at [0.01, 0.1, 1, 10, 100], we chose the radial basis function with gamma value of $\frac{1}{N_{\mathrm{features}}}=\frac{1}{42},$ other hyperparameters are at the default values.
  - Extreme gradient boosting: we tuned the hyperparameter of number of trees at [10, 20, 30, 40, 50, 60, 70, 80, 90, 100], other hyperparameters are at the default values.
  - Multilayer perceptron: We tuned the hyperparameter of hidden layer sizes at [50, 100, 200, 500, 1,000], other hyperparameters are at the default values.
2. The cluster: for each type of base learner, we try to compare two community detection methods.
  - Largest modularity using incremental greedy search (Blondel et al., 2008).
  - Multiple async label propagation (Raghavan et al., 2007).

### Performance Measures of Multilabel Learning

Evaluation of a multilabel learning model is not a trivial task (Zhang et al., 2015; Yuan et al., 2016; Zhang et al., 2017; You et al., 2018; Xiong et al., 2019; You et al., 2019). Inspired by the definition of Chou *et al.* (Chou, 2013) and practice of Madjarov et al. (2012), we utilized the following five metrics to evaluate the multilabel learning models throughout this work.

(16) $$\{\begin{array}{c} \mathrm{Aiming}=\frac{1}{N}\sum_{k=1}^{N}\left(\frac{\left\|L_{k}\cap L_{k}^{*}\right\|}{\left\|L_{k}^{*}\right\|}\right)\\ \mathrm{Coverage}=\frac{1}{N}\sum_{k=1}^{N}\left(\frac{\left\|L_{k}\cap L_{k}^{*}\right\|}{\left\|L_{k}\right\|}\right)\\ \mathrm{Accuracy}=\frac{1}{N}\sum_{k=1}^{N}\left(\frac{\left\|L_{k}\cap L_{k}^{*}\right\|}{\left\|L_{k}\cap L_{k}^{*}\right\|}\right)\\ \mathrm{Absolute}\;\mathrm{True}=\frac{1}{N}\sum_{k=1}^{N}\Delta (L_{k},L_{k}^{*})\\ \mathrm{Hamming}\;\mathrm{loss}=\frac{1}{N}\sum_{k=1}^{N}\left\|L_{k}⊖L_{k}^{*}\right\| \end{array}$$

where *N* is the total number of samples, *M* is the total number of labels, ⋃ represents union in set theory and ⋂ represents intersection in set theory, $L_{k}$ denotes the true label set of *k*-th sample, $L_{k}^{*}$ means the predicted label vector of *k*-th sample, ⊝ stands for the symmetric difference between two sets, and

(17) $$∆(L_{k},L_{k}^{*})=\{\begin{array}{c} 1,\mathrm{if}\;\mathrm{all}\;\mathrm{the}\;\mathrm{labels}\;\mathrm{in}\;L_{k}\;\mathrm{equal}\;L_{k}^{*}\\ 0,\mathrm{otherwise} \end{array}$$

In order to avoid the zero-divisor problem generated by all negative predictions, we add a pseudo-number 1 to 0 divisors in the calculation of the aiming metric. These above metrics have been used in a series of studies (Cheng et al., 2017a; Cheng et al., 2017b; Nanni and Brahnam, 2017).

### Performance Measures of Single-Label Learning

Apart from the metrics in the multilabel framework, we also utilized the following metrics to assess the single-label classification models.

(18) $$\{\begin{array}{c} \mathrm{Accuracy}=\frac{TP+TN}{TP+TN+FN+FP}\\ \mathrm{Specificity}=\frac{TN}{TN+FP}\\ \mathrm{Recall}=\frac{TP}{TP+FN}\\ F1=\frac{2TP}{2TP+FP+FN} \end{array}$$

where *TP*, *TN*, *FN*, and *TN* are true positives, true negatives, false positives, and false negatives for the prediction of each label, respectively. These metrics have widely been used in a large number of bioinformatics applications recently (Feng et al., 2017; Niu and Zhang, 2017; Sun et al., 2017; Wang et al., 2017; Xu et al., 2017; He et al., 2018; Li et al., 2018; Pan et al., 2018; Qiao et al., 2018; Xiong et al., 2018; Xu et al., 2018; Zhang et al., 2018; Bian et al., 2019; Wei et al., 2019a; Wei et al., 2019b; Zou et al., 2019). In addition, we also calculated the area under the receive operating characteristic curve (AUC) by the trapezoidal rule.

### Model Validation Method

There are mainly three methods to evaluate the generalization ability of a classification model, such as the independent testing method, *k*-fold cross validation, and the jackknife method. In order to fairly compare our proposed model with previous works on the same benchmark dataset, we utilized the jackknife method for the model validation in the multilabel learning framework. Jackknife is a resampling method for parameter estimation. The jackknife estimation of a parameter is constructed by calculating the parameter for each subsample omitting the *i*-th observation and then takes the mean value of these parameters as final estimation.

In the model validation of single-label analysis, we utilized 10 times repeated 10-fold cross validation (10 × 10-fold CV) method. In *k*-fold cross validation (CV), the sample set is randomly partitioned into *k* subsets with equal size. Of the *k* subsets, one subset is selected as the validation data for testing the model, and the remaining *k* − 1 subsets are used for training. The cross-validation process is then repeated *k* times (the folds), with each of the *k* subsets used exactly once as the validation data. The 10-fold cross-validation is proven to be a better alternative of jackknife method in terms of bias, variance, and computation complexity (Kohavi, 1995). We also repeated 10-fold CV 10 times in shuffled benchmark dataset to further reduce the estimation variance.

## Results and Discussion

### Label Correlation Analysis

**Figure 1:**
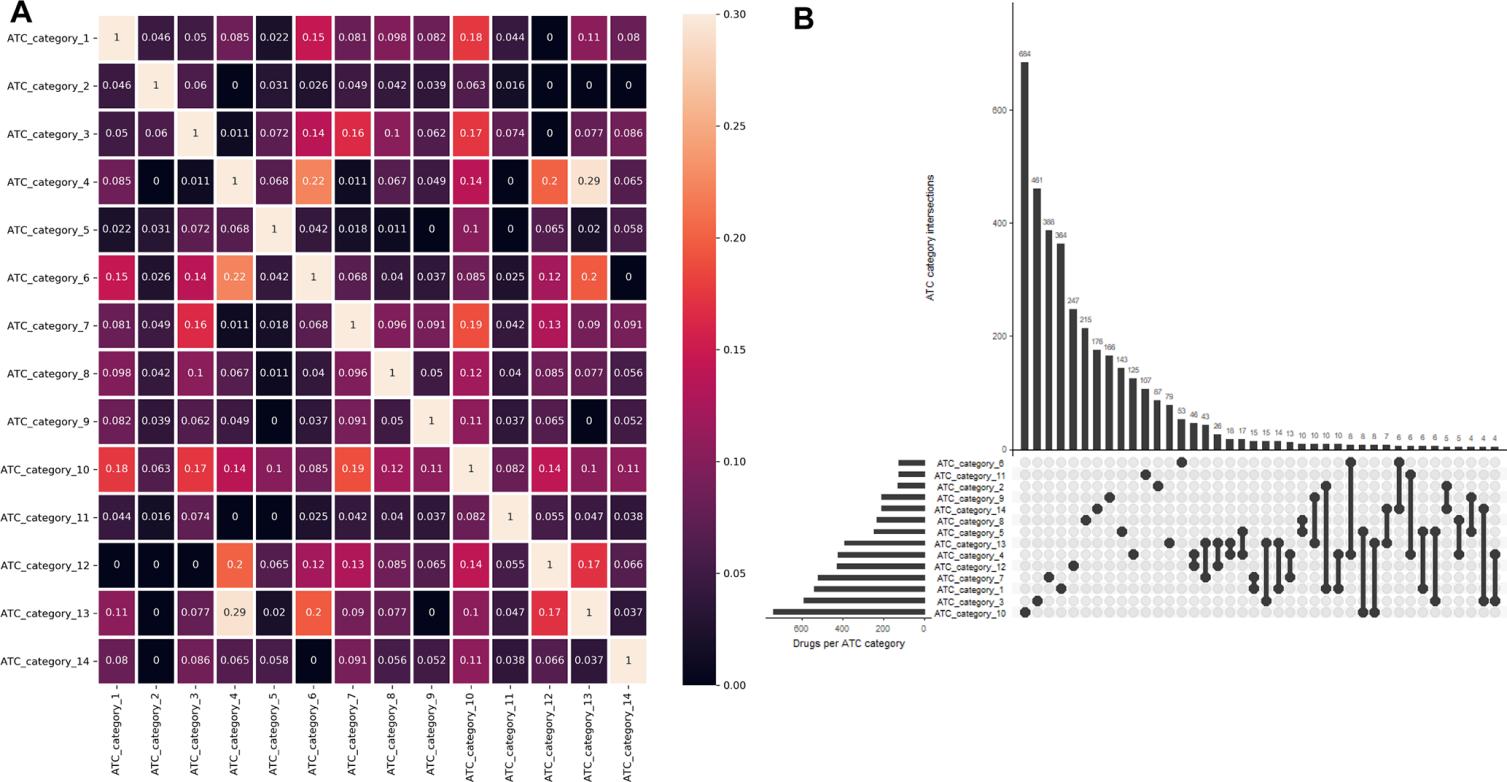
Label correlation landscape. **(A)** The pair wise visualization of Cramér’s V statistics for all the labels in a heatmap manner. **(B)** The UpSet visualization of label intersections. The horizontal bar shows the number of drugs per ATC category, and the vertical bar shows the number of drugs per ATC category intersection.

One major advantage of multilabel learning framework is the explicit exploitation of label correlations (Zhang and Zhou, 2014). We calculated bias corrected Cramér’s V statistics for all the label pairs and depicted them in a heatmap manner (**Figure 1A**), and the UpSet visualization of label intersections is depicted in **Figure 1B**. The results indicated that 46 drugs are both labeled as ATC category 4 (dermatologicals) and ATC category 12 (respiratory system), 43 drugs are both labeled as ATC category 13 (sensory organs) and ATC category 7 (anti-infectives for systemic use), which can be explained by the fact that many widely applied corticosteroids, such as dexamethasone, betamethasone, and fluocortolone, can be used both in dermatology and respirology medicine. We also found that several label sets are correlated, especially for ATC category 4 (dermatologicals) and ATC category 13 (sensory organs), of which the Cramér’s V statistic is 0.29. Details about the pairwise intersection numbers of drugs and the pairwise Cramér’s V statistics between all the labels are shown in **Table S1** and **Table S2**.

### Multilabel Performance Comparison

**Table 1** shows the prediction performances based on the jackknife test among different methods on the benchmark dataset. We found the absolute true value of almost all our NLSP-based methods performed better than that of other methods, which is the most stringent metric for multilabel learning. Among all the NLSP-based models, the NLSP-XGB-LPA performs the best, consistently better than all the other methods trained on benchmark dataset, in terms of aiming, coverage, accuracy, and absolute true. As for the value of absolute true, our NLSP-XGB-LPA has boosted ∼11.67% compared to the best deep learning model trained on the same benchmark dataset (Lumini and Nanni, 2018). As for the clusterer, we found that the LPA method performs consistently better than the Louvain method in all the NLSP-based models (**Figure S1**), so we append the suffix of “-LPA” to all the NLSP-based models. We then trained the final NLSP-XGB-LPA model on the full benchmark dataset using previous optimized hyperparameters. This model can be accessed through https://github.com/dqwei-lab/ATC.

**Table 1:** Comparison with other state-of-the-art multilabel predictors.

Method	DL^a^	Aiming	Coverage	Accuracy	Absolute true	Hamming loss
EnsANet_LR ⊕ DO^c^ (τ = 0.25) (Lumini and Nanni, 2018)	Yes	0.7957	0.8335	0.7778	0.7090	Not available
EnsANet_LR ⊕DO^c^ (τ = 0.5) (Lumini and Nanni, 2018)	Yes	0.9011	0.7162	0.7232	0.6871	
EnsLIFT (Nanni and Brahnam, 2017)	No	0.7818	0.7577	0.7121	0.6330	
iATC-mHyb^c^ (Cheng et al., 2017a)	No	0.7191	0.7146	0.7132	0.6675	
Chen et al. (Chen et al., 2012)	No	0.5076	0.7579	0.4938	0.1383	
iATC-mISF (Cheng et al., 2017b)	No	0.6783	0.6710	0.6641	0.6098	
NLSP-ERT-LPA	No	0.7948	0.7691	0.7578	0.7213	0.03817
NLSP-RF-LPA	No	0.8072	0.7889	0.7778	0.7489	**0.03427**
NLSP-SVM-LPA	No	0.7844	0.7529	0.7370	0.6925	0.04322
NLSP-XGB-LPA	No	**0.8135^b^**	**0.7950**	**0.7828**	**0.7497**	0.03429
NLSP-MLP-LPA	No	0.7958	0.7858	0.7591	0.7090	0.04032

a DL denotes whether this model is a deep learning-based method.

b The bold value stads for the best value of specific metrics.

c These models are trained on a modified benchmark dataset, whose metrics are not comparable to our model.

### Label Community Analysis

**Figure 2:**
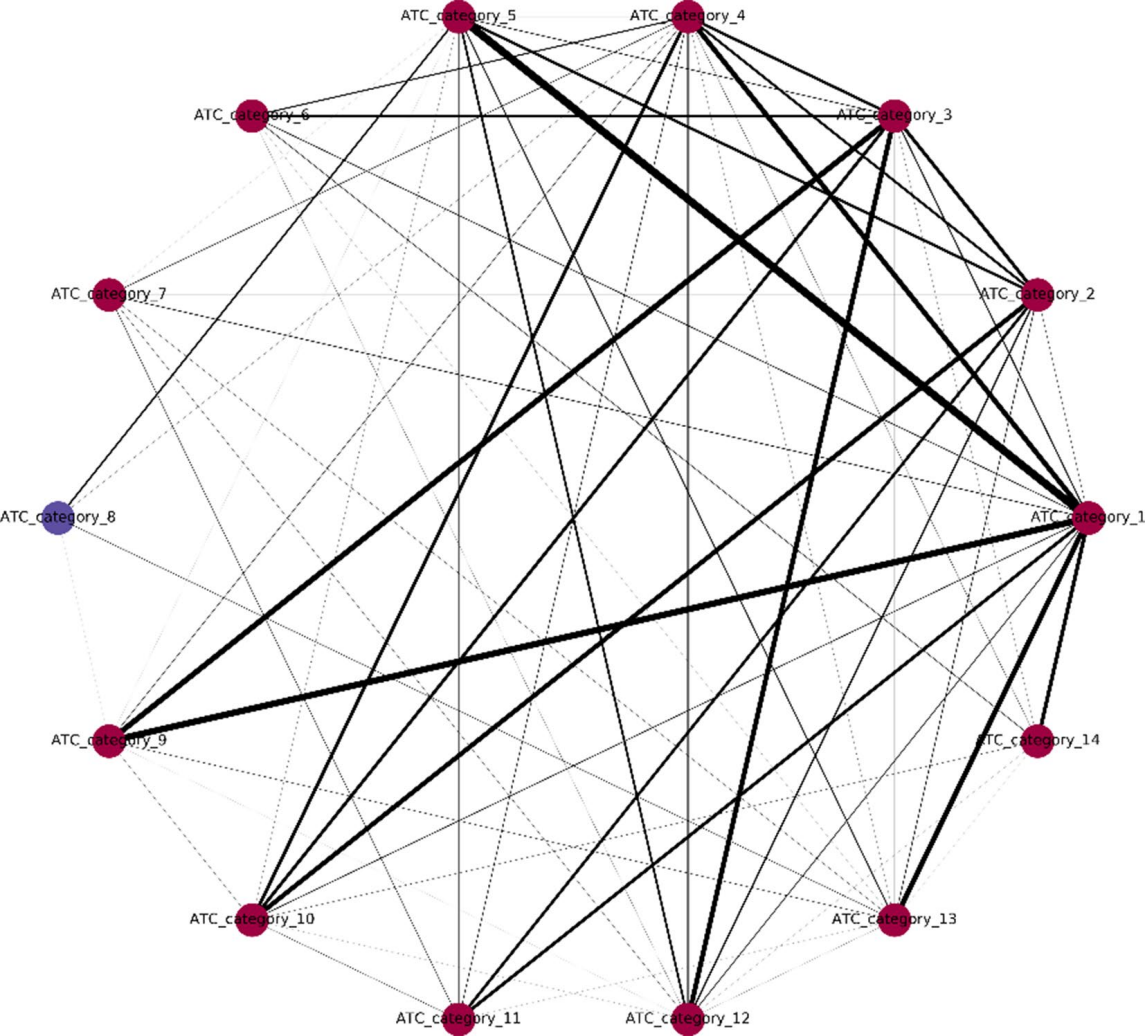
Label relation graph. Different colors stand for different communities. The line width represents the weight between two labels. Communities are detected by multiple async label propagation method, while the weight represents the frequency of label co-occurrence.

One major innovation of NLSP method is the construction of label relation graph, which is built on the concept of label co-occurrence (Szymanski and Kajdanowicz, 2019). The communities detected in the label relation graph will not only help to improve the classification performance but also provide us with deeper insights of the intrinsic label structure. We extracted the community membership information from the final model of NLSP-XGB-LPA (shown in **Figure 2**). We found that there are two communities detected, in which ATC category 8 (anti-infectives for systemic use) lies in a unique community. In terms of medicinal chemistry and clinical pharmacotherapeutics, anti-infectives for systemic use are structure variant and usage limited compared to other 16 types of drugs. For example, daptomycin (DB00080) is one of the anti-infectives for systemic use, which is composed of an unusual molecular structure of lipopeptide with limited indications for skin and skin structure infections caused by Gram-positive infections, *S. aureus* bacteremia, and right-sided *S. aureus* endocarditis (Henken et al., 2010). The community membership learnt from benchmark dataset is surprising but intuitive. This result suggests the potential pattern extraction power of network-based machine learning models in terms of pharmacology.

### Single-Label Analysis

Apart from multilabel learning metrics, it is often useful to evaluate multilabel learning models in a label-wise manner (Michielan et al., 2009; Mayr et al., 2016). We utilized the parameters of the best-performing model of NLSP-XGB-LPA and conducted 10 times repeated 10-fold cross-validation (10 × 10-fold CV) because the jackknife test is rather time consuming. The details are listed in **Table 2**. We found that our NLSP-XGB-LPA performs well in all the single-label subtasks of ATC prediction, especially for the label of “anti-infectives for systemic use,” reaching an AUC at 0.9946. Compared to a dedicated single-label classification system for cardiovascular system (Gurulingappa et al., 2009), our best-performing multilabel model boosted the value of accuracy from 0.8947 into 0.9490.

**Table 2:** Label-wise analysis of best-performing multilabel learning model.

Predictive label	Accuracy	Specificity	Recall	F1 score	AUC	Evaluation method
Alimentary tract and metabolism	0.9269	0.7312	0.7549	0.7406	0.9550	10 × 10-fold CV
Blood and blood forming organs	0.9793	0.7754	0.5644	0.6430	0.9493	10 × 10-fold CV
Cardiovascular system	0.9490	0.8371	0.8274	0.8306	0.9752	10 × 10-fold CV
Dermatologicals	0.9403	0.7966	0.6038	0.6845	0.9472	10 × 10-fold CV
Genitourinary system and sex hormones	0.9691	0.8148	0.6682	0.7294	0.9539	10 × 10-fold CV
Systemic hormonal preparations, excluding sex hormones and insulins	**0.9867^a^**	0.8227	0.7605	0.7816	0.9940	10 × 10-fold CV
Anti-infectives for systemic use	0.9793	**0.9276**	**0.9170**	**0.9215**	**0.9946**	10 × 10-fold CV
Antineoplastic and immunomodulating agents	0.9792	0.8683	0.7724	0.8126	0.9804	10 × 10-fold CV
Musculoskeletal system	0.9820	0.8707	0.7836	0.8209	0.9842	10 × 10-fold CV
Nervous system	0.9511	0.8581	0.8913	0.8733	0.9825	10 × 10-fold CV
Antiparasitic products, insecticides and repellents	0.9863	0.8312	0.7358	0.7714	0.9803	10 × 10-fold CV
Respiratory system	0.9573	0.8432	0.7516	0.7923	0.9720	10 × 10-fold CV
Sensory organs	0.9492	0.8206	0.6367	0.7140	0.9487	10 × 10-fold CV
Various	0.9717	0.7681	0.6997	0.7241	0.9703	10 × 10-fold CV
Cardiovascular system (Gurulingappa et al., 2009)	0.8947	Not available				100 × bootstrapping
Cardiovascular system (Gurulingappa et al., 2009)	0.7712					Test set
SuperPred (Dunkel et al., 2008)	0.676^b^					Jackknife

a The bold value stands for the best value of specific metrics.

b The mean accuracy of flattened 850 ATC classes.

## Conclusion

Based upon the NLSP method, we have achieved the state-of-the-art performance on the benchmark dataset using the similarity-based features such as chemical–chemical interaction and structural and fingerprint similarities of a compound to other compounds belonging to the different ATC categories. Label community and single-label analysis were also performed on the benchmark dataset. There are three major conclusions can be reached. First, compared to dedicated single-label models (Dunkel et al., 2008; Gurulingappa et al., 2009), multilabel learning framework could improve the performance on single-label metrics by incorporating label correlation information. Second, compared to feature engineering tricks (Nanni and Brahnam, 2017; Lumini and Nanni, 2018), the introduction of new method such as NLSP could generate more performance improvement. Third, at least in the ATC prediction task, the NLSP method, which adopts ideas from network research community and captures the correlation of labels in a data-driven manner, can perform better than the models based on deep learning techniques, especially in the absolute true rate metric. The idea behind NLSP method is fascinating, and the power of NLSP remains to be unleashed for the multilabel learning tasks in drug discovery.

Although the NLSP method was the first time to be applied to the multilabel classification task in pharmacology and achieved good performance in the preliminary results, there are shortcomings in several aspects in this study. First, the similarity-based features are not recalculated for the specific communities detected by the NLSP methods. Second, the rigidity of the model validation can be improved by the independent external dataset. Last but not the least, the number of communities detected by NLSP on this drug classification problem is too low, which may be not an ideal dataset for proving the predictive power of the NLSP-based method. These problems can be addressed in the further studies.

## Data Availability

All datasets generated for this study are included in the manuscript and/or **Supplementary Files**.

## Author Contributions

YX, D-QW and XW contributed conception and design of the study; XW and YW organized the database; XW, YW and ZX performed the statistical analysis; XW wrote the first draft of the manuscript; XW and YW wrote sections of the manuscript. All authors contributed to manuscript revision, read and approved the submitted version.

## Funding

This work was supported by the funding from National Key Research Program (contract no. 2016YFA0501703), National Natural Science Foundation of China (grant no. 31601074, 61872094, 61832019), and Shanghai Jiao Tong University School of Medicine (contract no. YG2017ZD14).

## Conflict of Interest Statement

The authors declare that the research was conducted in the absence of any commercial or financial relationships that could be construed as a potential conflict of interest.
