# Supplementary material for: ATC-NLSP: Prediction of the Classes of Anatomical Therapeutic Chemicals Using a Network-Based Label Space Partition Method
Source: Front Pharmacol. 2019 Sep 5;10:971. doi: 10.3389/fphar.2019.00971 (PMC6739564; doi:10.3389/fphar.2019.00971)
Supplement: Supplementary file 1 [file DataSheet_1.docx]

*Supplementary Material*

**ATC-NLSP: prediction of the classes of anatomical therapeutic chemicals using a network-based label space partition method**

Xiangeng Wang^#^, Yanjing Wang^#^, Zhenyu Xu, Yi Xiong^*^ and Dong-Qing Wei^*^

State Key Laboratory of Microbial Metabolism, School of Life Sciences and Biotechnology, Shanghai Jiao Tong University, Shanghai, China

^#^Equal contribution

^*^ Correspondence:

Yi Xiong

[xiongyi@sjtu.edu.cn](mailto:xiongyi@sjtu.edu.cn)

Dong-Qing Wei

[dqwei@sjtu.edu.cn](mailto:dqwei@sjtu.edu.cn)

| **Table S1:** The pair-wise intersection numbers of drugs on the benchmark dataset | | |
| --- | --- | --- |
| ATC_category | ATC_category | Number of intesction |
| 13 | 4 | 149 |
| 12 | 4 | 123 |
| 13 | 12 | 106 |
| 13 | 1 | 98 |
| 4 | 1 | 95 |
| 13 | 3 | 93 |
| 13 | 7 | 89 |
| 4 | 3 | 73 |
| 7 | 4 | 65 |
| 12 | 1 | 65 |
| 12 | 3 | 64 |
| 6 | 4 | 62 |
| 3 | 1 | 56 |
| 6 | 3 | 54 |
| 13 | 6 | 54 |
| 6 | 1 | 53 |
| 5 | 4 | 48 |
| 12 | 6 | 41 |
| 7 | 1 | 34 |
| 2 | 1 | 31 |
| 5 | 1 | 25 |
| 7 | 5 | 25 |
| 13 | 10 | 25 |
| 13 | 9 | 23 |
| 13 | 5 | 17 |
| 4 | 2 | 16 |
| 10 | 3 | 16 |
| 10 | 4 | 14 |
| 12 | 2 | 14 |
| 13 | 2 | 14 |
| 9 | 5 | 13 |
| 11 | 4 | 13 |
| 12 | 10 | 13 |
| 5 | 3 | 12 |
| 9 | 3 | 11 |
| 11 | 5 | 11 |
| 8 | 5 | 10 |
| 14 | 13 | 10 |
| 10 | 1 | 9 |
| 9 | 4 | 8 |
| 10 | 5 | 8 |
| 10 | 2 | 7 |
| 12 | 5 | 7 |
| 14 | 2 | 7 |
| 11 | 1 | 6 |
| 11 | 7 | 6 |
| 14 | 6 | 6 |
| 7 | 2 | 5 |
| 8 | 4 | 5 |
| 3 | 2 | 4 |
| 12 | 9 | 4 |
| 14 | 1 | 4 |
| 14 | 3 | 4 |
| 14 | 4 | 4 |
| 14 | 12 | 4 |
| 9 | 1 | 3 |
| 12 | 7 | 3 |
| 5 | 2 | 2 |
| 10 | 9 | 2 |
| 13 | 11 | 2 |
| 14 | 10 | 2 |
| 9 | 8 | 1 |
| 11 | 2 | 1 |
| 11 | 10 | 1 |
| 12 | 11 | 1 |
| 13 | 8 | 1 |

| **Table S2:** The pair-wise Cramér's V statistics between all the labels on the benchmark dataset | | | | | | | | | | | | | | |
| --- | --- | --- | --- | --- | --- | --- | --- | --- | --- | --- | --- | --- | --- | --- |
|  | ATC_category_1 | ATC_category_2 | ATC_category_3 | ATC_category_4 | ATC_category_5 | ATC_category_6 | ATC_category_7 | ATC_category_8 | ATC_category_9 | ATC_category_10 | ATC_category_11 | ATC_category_12 | ATC_category_13 | ATC_category_14 |
| ATC_category_1 | 0.9989 | 0.0458 | 0.0505 | 0.0849 | 0.0219 | 0.1463 | 0.0811 | 0.0983 | 0.0824 | 0.1756 | 0.0438 | 0 | 0.1063 | 0.0799 |
| ATC_category_2 | 0.0458 | 0.9961 | 0.0603 | 0 | 0.0311 | 0.0261 | 0.0491 | 0.0417 | 0.0386 | 0.0625 | 0.0163 | 0 | 0 | 0 |
| ATC_category_3 | 0.0505 | 0.0603 | 0.999 | 0.0109 | 0.0723 | 0.138 | 0.165 | 0.1041 | 0.0622 | 0.1742 | 0.0742 | 0 | 0.0774 | 0.0859 |
| ATC_category_4 | 0.0849 | 0 | 0.0109 | 0.9987 | 0.068 | 0.2231 | 0.011 | 0.0668 | 0.0491 | 0.1372 | 0 | 0.2011 | 0.2923 | 0.0652 |
| ATC_category_5 | 0.0219 | 0.0311 | 0.0723 | 0.068 | 0.9978 | 0.0419 | 0.0179 | 0.0105 | 0 | 0.1024 | 0 | 0.0646 | 0.0204 | 0.0581 |
| ATC_category_6 | 0.1463 | 0.0261 | 0.138 | 0.2231 | 0.0419 | 0.9959 | 0.0681 | 0.04 | 0.037 | 0.0853 | 0.0249 | 0.1228 | 0.1969 | 0 |
| ATC_category_7 | 0.0811 | 0.0491 | 0.165 | 0.011 | 0.0179 | 0.0681 | 0.9989 | 0.0963 | 0.0906 | 0.1889 | 0.0418 | 0.1289 | 0.0895 | 0.0913 |
| ATC_category_8 | 0.0983 | 0.0417 | 0.1041 | 0.0668 | 0.0105 | 0.04 | 0.0963 | 0.9977 | 0.0502 | 0.1196 | 0.0402 | 0.0854 | 0.0772 | 0.0558 |
| ATC_category_9 | 0.0824 | 0.0386 | 0.0622 | 0.0491 | 0 | 0.037 | 0.0906 | 0.0502 | 0.9975 | 0.1067 | 0.0372 | 0.0652 | 0 | 0.0521 |
| ATC_category_10 | 0.1756 | 0.0625 | 0.1742 | 0.1372 | 0.1024 | 0.0853 | 0.1889 | 0.1196 | 0.1067 | 0.9992 | 0.0819 | 0.1409 | 0.1048 | 0.1076 |
| ATC_category_11 | 0.0438 | 0.0163 | 0.0742 | 0 | 0 | 0.0249 | 0.0418 | 0.0402 | 0.0372 | 0.0819 | 0.9959 | 0.0554 | 0.0467 | 0.0376 |
| ATC_category_12 | 0 | 0 | 0 | 0.2011 | 0.0646 | 0.1228 | 0.1289 | 0.0854 | 0.0652 | 0.1409 | 0.0554 | 0.9987 | 0.1707 | 0.066 |
| ATC_category_13 | 0.1063 | 0 | 0.0774 | 0.2923 | 0.0204 | 0.1969 | 0.0895 | 0.0772 | 0 | 0.1048 | 0.0467 | 0.1707 | 0.9986 | 0.0371 |
| ATC_category_14 | 0.0799 | 0 | 0.0859 | 0.0652 | 0.0581 | 0 | 0.0913 | 0.0558 | 0.0521 | 0.1076 | 0.0376 | 0.066 | 0.0371 | 0.9975 |


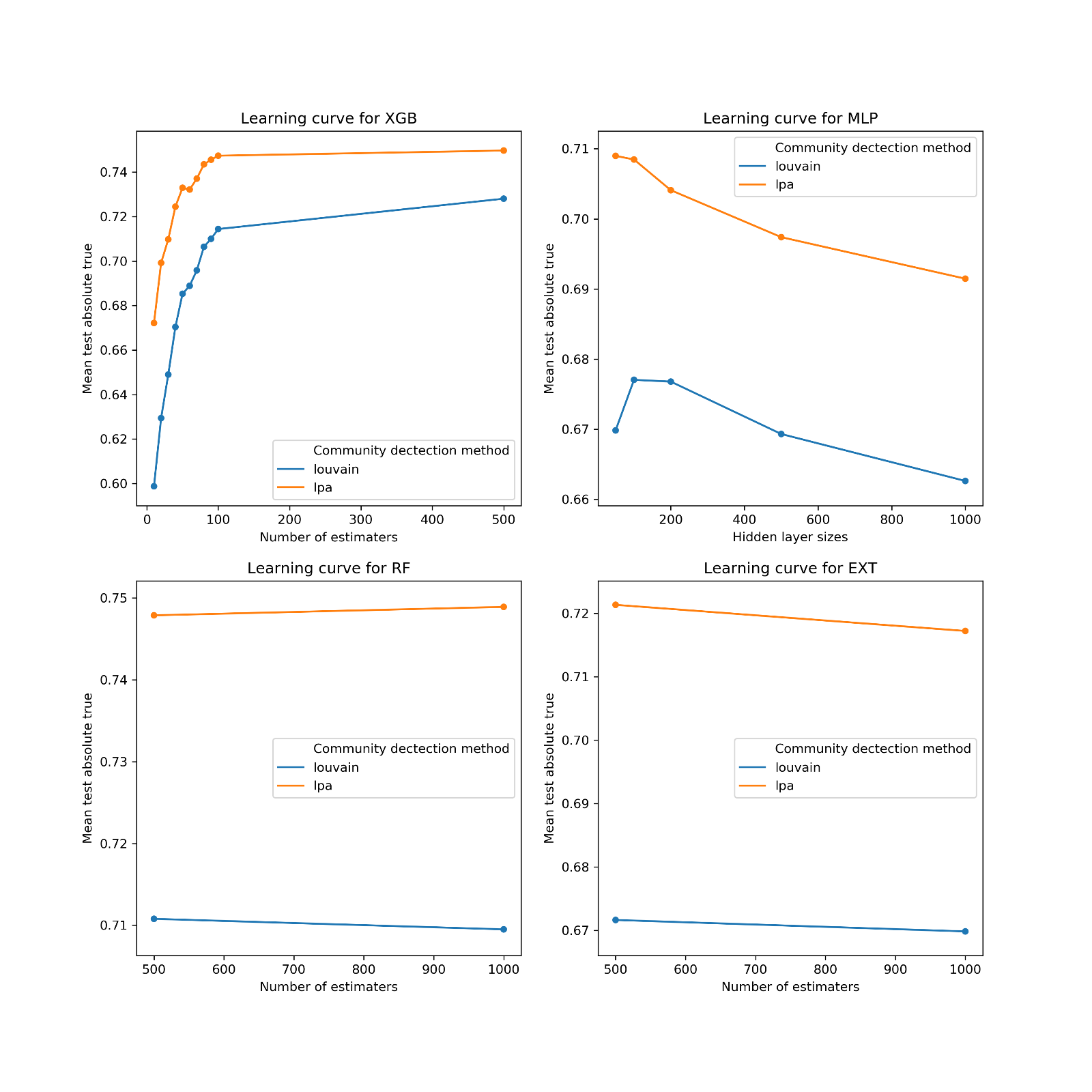


**Figure S1:** Learning curves for base learners of XGB, MLP, RF and EXT. The LPA community detection method performs consistently better than Louvain method on all the base learners.

**Method S1:** Largest modularity using incremental greedy search

The method of largest modularity using incremental greedy search is based on greedy aggregation of communities, beginning with communities with single convex and merging the communities iteratively. In each step, two communities are merged when the merging makes the highest contribution to modularity. The algorithm halts when there is no merge that could increase current modularity. This method is frequently referred as “Louvain method” in the network research community. (Blondel et al. 2008). This method is proven to have linear time complexity on typical and sparse data.

In the first phase, we begin with a weighted network of *N* nodes, and each node we assign different community label. We thus have *N* communities in the first partition. Then, for each node *i* we evaluate the gain of modularity for each neighbour *j* if we remove *i* from its community and place it into the community of *j*. The node *i* is then incorporated in the community where the gain of modularity is maximum, at the requirement of the positive gain. If no positive gain detected, the node *i* stays static. This kind of process continues iteratively and sequentially for all nodes until no more gains of modularity possible. In a word, the first phase has reached a local optimum of the modularity. The gain in modularity $\Delta Q$ by moving a node *i* into a community$C$ can be calculated by

$$\Delta Q=\left[ \frac{\sum_{\mathrm{in}} +2k_{i,\mathrm{in}}}{2m}-(\frac{\sum_{\mathrm{tot}} +k_{i}}{2m})^{2} \right]-[\frac{\sum_{\mathrm{in}}}{2m}-(\frac{\sum_{\mathrm{tot}}}{2m})^{2}-(\frac{k_{i}}{2m})^{2}]$$

where $\sum_{\mathrm{in}}$is the sum of the weights of the links inside $C$, $\sum_{\mathrm{tot}}$is the sum of the weights of the links incident to nodes in $C$, $k_{i}$is the sum of the weights of the links incident to node *i*, $k_{i,\mathrm{in}}$is the sum of the weights of the links from *i* to nodes in $C$ and *m* is the sum of the weights of all the links in the network.

In the second phase, a new network, whose nodes are the communities detected in the first phase are built. The weights of the links between two new nodes are the sum of the links between nodes in the original communities. Self-loops emerged due to the links between the nodes of the same community. We define the combination of these two phases as a pass. The passes are iterated until a maximum of modularity is obtained. The number of communities decreases at each pass, and most computing time is spent in the first pass.

**Method S2:** Multiple async label propagation

The detailed process of multiple async label propagation can be summarized as follows (Raghavan et al. 2007),

1. Initialize the labels at all nodes in the network. For a given node $x$, $C_{x}\left( 0 \right)=x$, where $C_{x}(t)$ stands for the label of node *x* at time *t*.
2. Set $t=1.$
3. Arrange the nodes in the network in a random sequence and set it to X.
4. For each $x\in X$ selected in a specific order, let $C_{x}\left( t \right)=f(C_{x_{i1}}\left( t \right),\ldots,C_{x_{im}}\left( t \right),C_{x_{i\left( m+1 \right)}}\left( t-1 \right),\ldots,C_{x_{ik}}(t-1))$, where $f$ returns the label with the highest frequency among neighbors and ties are broken in a uniform and random manner, $x_{i1},\ldots,x_{im}$ are neighbors of *x* that have already been updated in the current iteration while $x_{i(m+1)},\ldots,x_{ik}$ stand for neighbors are not yet updated in current iteration. It is the reason why this algorithm is called “async”.
5. If every node has a label that the maximum number of their neighbors have, then stop the algorithm. Else, set $t=t+1$ and go to (iii).

This algorithm is proven to have a near-linear time complexity of $O(M+N)$, where $N$ is the number of nodes and $M$ is the number of edges.

**References**

Blondel VD, Guillaume J-L, Lambiotte R, Lefebvre E. 2008. Fast unfolding of communities in large networks. Journal of statistical mechanics: theory and experiment.2008:P10008.

Raghavan UN, Albert R, Kumara S. 2007. Near linear time algorithm to detect community structures in large-scale networks. Physical review E.76:036106.
